# Beyond Daily Values: Are Day-to-Day and Albumin-Adjusted Ratios of IL-6, PCT, and CRP Better Predictors of Ventilator-Associated Pneumonia?

**DOI:** 10.3390/life15111697

**Published:** 2025-11-01

**Authors:** Tobias Bexten, Golo-Sung Haarmeier, Johann Klein, Holger A. Lindner, Chaimae Rahali, Verena Schneider-Lindner

**Affiliations:** 1 Department for Interdisciplinary Intensive Medicine and Intermediate Care, Helios Dr. Horst Schmidt Hospital Wiesbaden, 65199 Wiesbaden, Germany; chaimae.rahali@helios-gesundheit.de; 2 Department of Internal Intensive Care Medicine, Nuremberg Hospital, 90419 Nuremberg, Germany; golo-sung.haarmeyer@klinikum-nuernberg.de; 3 Department for Neurosurgery, Helios Dr. Horst Schmidt Hospital Wiesbaden, 65199 Wiesbaden, Germany; johann.klein@helios-gesundheit.de; 4 Department of Anesthesiology, Intensive Care Medicine and Pain Medicine, Medical Faculty Mannheim, Heidelberg University, 68167 Mannheim, Germany; holgera.lindner@medma.uni-heidelberg.de (H.A.L.); verena.schneider-lindner@medma.uni-heidelberg.de (V.S.-L.)

**Keywords:** ventilator-associated pneumonia, IL-6, CRP, procalcitonin, biomarkers, albumin, albumin ratio, ICU

## Abstract

Background: Ventilator-associated pneumonia (VAP) is a frequent complication in neurosurgical intensive care patients. This leads to prolonged mechanical ventilation, increased 30-day mortality rates, and extended hospital stays. However, early diagnosis remains a challenge. Biomarkers, such as IL-6, PCT, and CRP, are considered a cornerstone for recognizing VAP and initiating early treatment. Only a very limited number of studies have compared IL-6, PCT, and CRP, focusing on day-to-day dynamics and their albumin ratios. Therefore, we investigated whether, compared with their daily levels, the day-to-day dynamics and albumin-adjusted ratios of IL-6, PCT, and CRP offer improved diagnostic value for VAP. Second, we investigated these biomarkers in patients treated for VAP who did not meet the criteria for VAP. Methods: In this exploratory, matched case–control study, we investigated 171 neurosurgical ICU patients. Daily biomarker levels, their dynamics, and ratios to serum albumin were assessed beginning four days before VAP. Logistic regression and receiver operating curve (ROC) analyses were performed to evaluate the association between each biomarker and VAP. Results: IL-6 and its day-to-day dynamics demonstrated the largest differences between VAP patients and nonVAP patients (*r* = 0.631; *r* = 0.452) and were associated with VAP, yielding AUCs of 0.816 and 0.726, respectively. In contrast, for PCT, we did not demonstrate any associative utility, whereas CRP showed a significant, moderate effect size on the day of VAP occurrence (*p* = 0.015 *; *r* = 0.351). We could not demonstrate any superiority in the day-to-day dynamics or the albumin-adjusted ratios compared to the daily values. For patients who were treated for VAP without fulfilling the criteria for biomarkers, we did not observe any significant difference from nonVAP patients.

## 1. Introduction

Ventilator-associated pneumonia (VAP) is clinically characterized by new, persistent, or progressive infiltrates on chest X-rays, accompanied by two of the following criteria: (1) white blood cell count >10/nL or <4/nL, (2) fever >38 °C, or (3) purulent secretion [1]. Aside from burn patients, neurosurgical patients have the highest rate of VAP [2], which leads to prolonged mechanical ventilation, increased 30-day mortality rates, and extended hospital stays [3]. Moreover, it incurs additional costs of up to 40,000 US dollars per case, making it the second-most-expensive hospital-acquired infection [4].

In addition to clinical criteria, biomarkers are considered a cornerstone for recognizing VAP and initiating early treatment.

Procalcitonin (PCT) and C-reactive protein (CRP) are frequently used biomarkers in intensive care settings [5]. In some hospitals, IL-6 is also routinely used for this purpose [6]. Their clinical utility differs depending on their response time to an inflammatory stimulus and the extent of their increase [7,8,9]. While IL-6 is one of the fastest biomarkers for inflammation, peaking at 6 h after the stimulus [10], CRP reaches the maximum level with a delay of 36–50 h [11,12]. PCT responds at 4–6 h after exposure and peaks at 12–24 h [13].

For surgical and medical patients, the CRP and PCT levels might indicate intensive care unit (ICU) patients at risk of developing VAP 72 h after intubation [14,15].

Compared with other cytokines, IL-6 levels increase 24–48 h before clinical evidence of an inflammatory process is observed [7]. In a previous study of neurosurgical ICU patients, we showed that IL-6 levels increased 24 h before the diagnosis and treatment of VAP [16]. Among multiple biomarkers, IL-6 is the only one that distinguishes microbiologically confirmed VAP from suspected VAP [17].

Another difficulty in interpreting biomarkers is that their values vary widely across patients [18]. Therefore, day-to-day ratios might equalize these differences and thus might be more stable. In association with VAP, the CRP and PCT day-to-day dynamics were also tested, demonstrating superiority over CRP or PCT alone [19].

Other investigators have studied the predictive value of the CRP/albumin, PCT/albumin, and IL-6/albumin ratios, focusing on 30-day mortality rates and acute kidney injury [20,21,22]. Since most inflammatory markers, especially IL-6, downregulate albumin synthesis in the liver, their ratio might be of interest as a composite marker for VAP.

Thus, we hypothesized that day-to-day ratios rather than their daily values, as well as their albumin ratio, could further improve their performance as classifiers of VAP in neurosurgical ICU patients.

Second, we differentiated VAP cases into microbiologically confirmed cases (mcVAPs) and suspected but not microbiologically confirmed cases (suspVAPs) and assessed potential differences in biomarker levels between these groups in a secondary analysis.

Lastly, we included patients who received VAP treatment but did not meet the diagnostic criteria. Here, we compared biomarker values and clinical values to those of nonVAP patients. To address these questions, we designed this exploratory proof-of-concept study.

## 2. Materials and Methods

**Figure A1:**
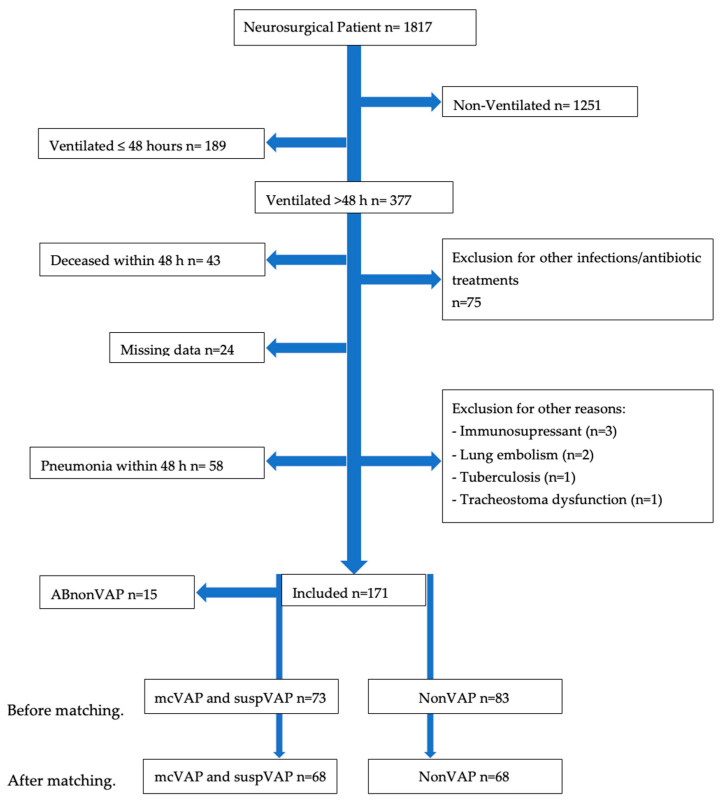
Study flow diagram.

Neurosurgical patients admitted to the ICU of Helios Dr. Horst Schmidt Hospital, either through the emergency department or postoperatively after major neurosurgical procedures, were included in this study. The observation period spanned from 1 January 2020 to 31 December 2023. Patients meeting the following criteria were excluded: younger than 18 years; extubation or death within the first 48 h of ICU admission; incomplete baseline data on hours of ventilation, antibiotic treatment, or radiological confirmation; and infection or aspiration pneumonia on admission. Additionally, we excluded patients who received antibiotic treatment at admission or within the first day after admission, as well as those who received antibiotics for other infections within the observation time (Appendix A Figure A1, study flow diagram).

Ventilator-associated pneumonia (VAP) is defined as mechanical ventilation for at least 48 h, accompanied by new, persistent, or progressive infiltrates on chest X-rays, along with at least two of the following criteria: (1) white blood cell count > 10,000 or <4000/μL, (2) fever > 38 °C, or (3) purulent respiratory secretions. Additionally, as the CDC proposed, specific antibiotic treatment is mandatory.

We divided the VAP patients into microbiologically confirmed (mcVAP) and nonmicrobiologically confirmed (suspVAP) groups.

We matched controls among VAP-free patients one-to-one based on the number of ventilator days. For this purpose, we defined the day of VAP as described above and matched controls who underwent ventilation for at least this duration. Additionally, we compared groups with identical timeframes where VAP might have occurred.

From January 2020 to April 2022, serum IL-6 was the preferred daily marker; thereafter, PCT and CRP were recorded daily. Therefore, we compared two groups and matched within each group.

In addition to our cases and controls, we identified a third group, comprising patients who received a directed antibiotic for VAP without formally fulfilling the VAP definition (ABnonVAP).

The overall observation period began at admission and lasted up to 20 days postadmission or until discharge or death, whichever occurred first. The days before VAP diagnosis and the day of diagnosis were consecutively numbered as days −4 to −1 and 0, respectively. For the control group, day 0 was defined as the day corresponding to the one-to-one VAP match. For patients assigned to the ABnonVAP group, day 0 was defined as the day we started antibiotic treatment. The observation period from day −4 to day 0 was chosen to capture the dynamics of the biomarkers in the days preceding the clinical diagnosis of VAP. This period was expected to reflect the early pathophysiological changes preceding infection.

Ethics: The study was approved by Ethics Committee II of the Medical Faculty Mannheim, University of Heidelberg (Reference number: 2024–821).

Statistical analysis: We first examined the daily values of each inflammatory biomarker (IL-6, CRP, and PCT) from day −4 to day 0. We subsequently assessed their changes by calculating ratios between consecutive days (days −3/−4, −2/−3, −1/−2, and 0/−1) and their albumin ratios.

We then tested for differences, including effect sizes, and conducted logistic regression analyses, reporting odds ratios, and, where appropriate, receiver operating characteristic (ROC) curves. Metric variables are indicated as the mean [±SD] and/or median [IQR]. As needed, the group differences were tested with Student’s *t* test or the Mann–Whitney U test for metric variables and the chi-square test for categorical variables. Cohen’s d (d), rank-biserial correlation (r), or Cramer’s V (V) were used to determine the corresponding effect size [23]. Receiver operating characteristic (ROC) curves were used to determine the area under the ROC (AUC), sensitivity, and specificity for each parameter in predicting VAP, including the odds ratio (OR) and 95% confidence interval (CI). Statistical significance was assumed at an alpha level of 0.05 and indicated by an asterisk (*). To account for multiple testing, we applied the Benjamini–Hochberg correction to the biomarker *p*-values. Statistical analysis was performed with JASP 0.18.2, and matching and graph visualization were performed with Julius.ai (Caesar Labs, Inc., San Francisco, CA, USA).

## 3. Results

### 3.1. Population

A total of 1817 neurosurgical patients admitted to the ICU were screened for eligibility. Of these, 377 patients were ventilated for >48 h. The details are provided in the Appendix A Figure A1. After all the exclusion criteria were applied, 171 patients were eligible for further analysis, with 73 patients fulfilling the criteria for VAP, 83 patients in the nonVAP group, and 15 patients in the ABnonVAP group; after matching, 68 VAP patients, 45 of whom had mcVAP and 23 of whom had suspVAP, with a drop-out rate of 6.4% after matching, were included.

Appendix A Table A1 provides the matching details and quality checks, and Appendix A Table A2 shows the distributions between the IL-6 and PCT/CRP groups.

### 3.2. Baseline Mcvap/Suspvap vs. Nonvap

Age and SAPS scores are provided in Table 1. Significantly more men than women were diagnosed with mcVAP/suspVAP (69.1% vs. 30.9%, X^2^(1) = 6.795, *p* < 0.009, V = 0.224).

No significant differences were observed between the mcVAP/suspVAP and nonVAP groups for admission age, SAPS, diagnosis, comorbidities, or operative procedures.

Similarly, there were no significant differences in IL-6, WBC, PCT, CRP, the P/F ratio, or body temperature at admission between the groups. The details are provided in Table 2. Significant differences in length of stay and hours of ventilation were noted between nonVAP and mcVAP/suspVAP patients (length of stay: 399.81 h [SD 360.13] vs. 599.46 h [SD 335.57], *p* < 0.001, r = 0.576; hours of ventilation: 198.49 h [SD 165.23] vs. 380.10 h [SD 225.32], *p* < 0.001, r = 0.920).

### 3.3. Biomarker Values vs. Day-To-Day Ratios and Ratios to Albumin to Predict VAP

Differences in biomarker levels between mcVAP and suspVAP: During the observation period from day −4 to day 0, we did not detect differences in IL-6, CRP, or PCT levels between the mcVAP and suspVAP groups. Therefore, we combined mcVAP and suspVAP into a single group for the following analysis.

IL-6: Daily Levels: Median IL-6 levels were significantly higher in mcVAP/suspVAP patients than in nonVAP patients from day −1 to day 0.

On day −1, the median IL-6 level was 63.10 pg/mL (IQR: 71.00) in the mcVAP/suspVAP group and 46.35 pg/mL (IQR: 74.43) in the nonVAP group (*p* < 0.04, r = 0.255).

The greatest difference was observed on day 0, with median IL-6 levels of 109 pg/mL (IQR: 180.25) in the mcVAP/suspVAP group and 35.40 pg/mL (IQR: 35.80) in the nonVAP group (*p* < 0.001, *r* = 0.631).

For the day-to-day ratios, we observed significant differences in the IL-6 day-to-day ratios on days −1/−2 and 0/−1. On days −1/−2, the ratio was 1.84 (IQR: 1.92) in the mcVAP/suspVAP group and 0.93 (IQR: 1.42) in the nonVAP group (*p* = 0.013, r = 0.313). On days 0 and 1, the ratios were 1.42 (IQR: 1.85) and 0.74 (IQR: 0.81), respectively (*p* < 0.001, r = 0.452).

IL-6-to-albumin quotient: IL-6-to-albumin ratios were significantly greater in mcVAP/SuspulentVAP patients than in nonVAP patients on day 0, with borderline significance on day −1. On day −1, the ratio was 22.77 (IQR: 37.56) vs. 12.09 (IQR: 14.72) (*p* < 0.054, r = 0.257). On day 0, the ratio was 41.48 (IQR: 65.07) in the mcVAP/suspVAP group vs. 12.09 (IQR: 14.72) in the nonVAP group (*p* < 0.001, r = 0.257).

**Figure 1:**
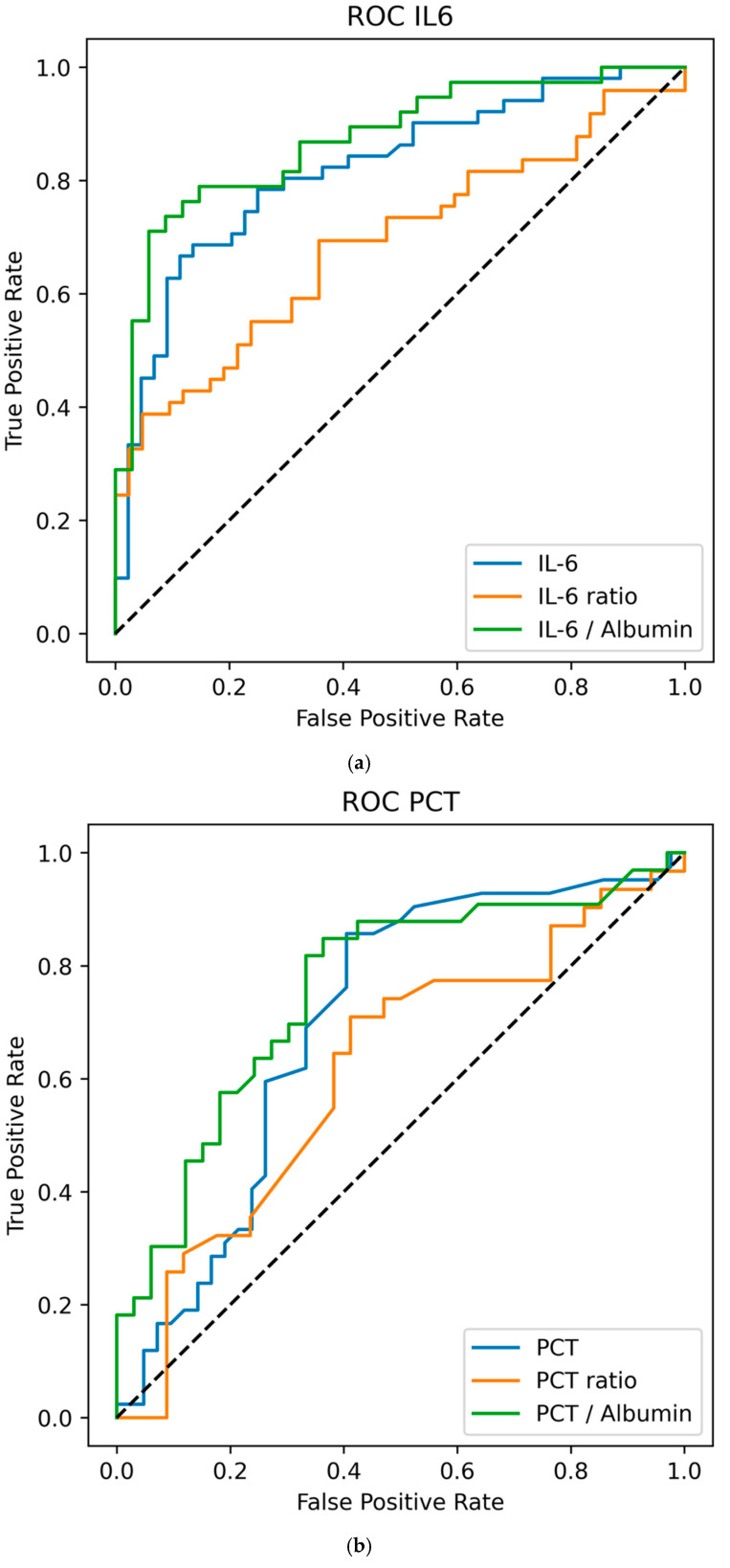

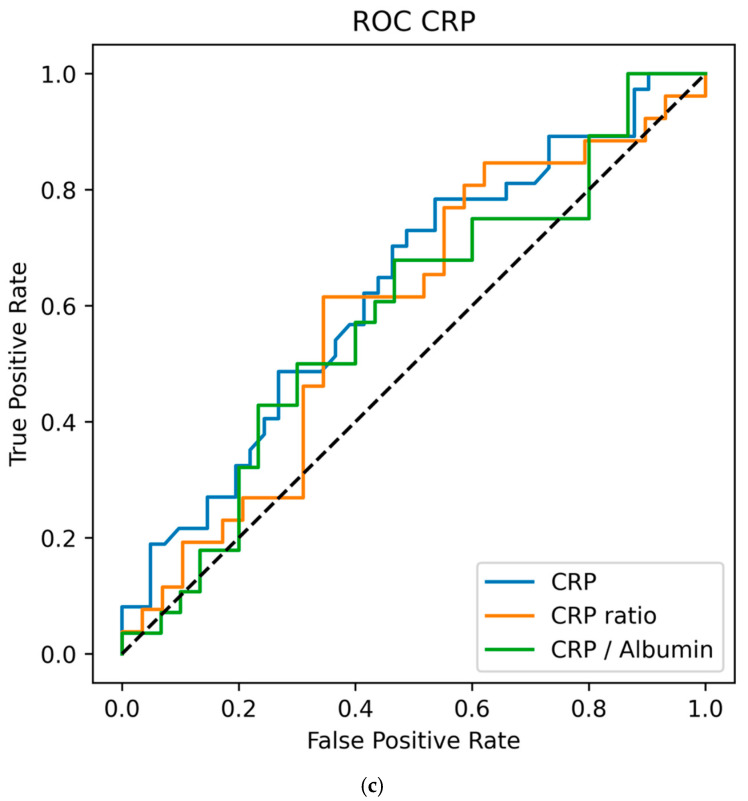
(**a**) ROC for IL-6 in VAP patients on day 0. Receiver operating characteristic (ROC) curves. IL-6 D0: Threshold = 26.26, sensitivity = 66.7%, specificity = 88.6%, AUC = 0.816. IL-6 D0/−1 ratio: threshold = 1.75, sensitivity = 38.8%, specificity = 95.2%, AUC = 0.726. IL-6/albumin D0: Threshold = 26.26, sensitivity = 71.1%, specificity = 94.1%, AUC = 0.864. (**b**) ROC for PCT in VAP patients on day 0. Receiver operating characteristic (ROC) curves for PCT. PCT D0: Threshold = 0.13, sensitivity = 85.7%, specificity = 59.5%; AUC = 0.707. PCT D0/−1 ratio: threshold = 0.90, sensitivity = 71.0%, specificity = 58.8%, AUC = 0.626 PCT/albumin D0: threshold = 0.04, sensitivity = 81.8%, specificity = 66.7%, AUC = 0.746. (**c**) ROC for CRP in VAP patients on day 0. ROC for CRP. CRP D0: Threshold = 8.50, sensitivity = 78.4%, specificity = 46.3%, AUC = 0.676. CRP D0/−1 ratio: Threshold = 1.05, sensitivity = 61.5%, specificity = 65.5%, AUC = 631. CRP/albumin D0: Threshold = 3.76, sensitivity = 67.9%, specificity = 53.3%, AUC = 636.

Logistic regression revealed that the IL-6 level on day 0 and the day 0/−1 ratio were significantly associated with VAP. The corresponding odds ratios (ORs) were 1.012 (*p* < 0.001, 95% CI: 1.004–1.020) for IL-6 on day 0 and 2.66 (*p* < 0.001, 95% CI: 1.390–5.091) for the day −1/−2 ratio, with AUCs of 0.816 and 0.726, respectively. The IL-6-to-albumin ratio significantly predicted VAP on day 0, with an OR of 1.065 (*p* < 0.001, 95% CI: 1.024–1.109) and an AUC of 0.864. Details are provided in Table 3, and the receiver operating curves are provided in Figure 1a.

PCT: Daily PCT levels were significantly different on day 0, with a median level of 0.24 ng/mL (IQR: 0.34) in the mcVAP/suspVAP group vs. 0.11 ng/mL (IQR: 0.19) in the nonVAP group (*p* = 0.004, r = 0.414). Day-to-day ratios: No statistically significant differences were found in the PCT day-to-day ratios from day −4 to day 0. PCT-to-albumin quotient: PCT-to-albumin ratio on day 0 was significantly different, with a median ratio of 0.08 (IQR: 0.14) in the mcVAP/suspVAP group and 0.04 (IQR: 0.05) in the nonVAP group (*p* = 0.004, r = 0). Logistic regression: In the logistic regression analysis, we did not identify significant differences in day levels, day-to-day ratios, or PCT-to-albumin ratios.

Table 1 provides further details, and the receiver operating curves are given in Figure 1b.

CRP: Daily CRP levels were significantly different on day 0, with a median level of 12.85 mg/L (IQR: 8.55) in the mcVAP/suspVAP group vs. 7.40 mg/L (IQR: 7.95) in the nonVAP group (*p* = 0.015, r = 0.351). Day-to-day ratios: No statistically significant differences were found for the CRP day-to-day ratios from day −4 to day 0. CRP-to-albumin quotient: In terms of the CRP-to-albumin ratio, we did not detect any statistically significant differences. Logistic regression analysis revealed that CRP values on day 0 were associated with VAP, with an OR of 1.093 (*p* = 0.012, 95% CI: 1.009–1.183) and an AUC of 0.676.

The details are provided in Table 4, and the receiver operating curves are provided in Figure 1c.

Adjustment for multiple testing: To control for multiple testing in biomarker analyses, we applied the Benjamini–Hochberg procedure (false discovery rate of 5%) to all biomarker-related *p*-values. This family included all tests comparing IL-6, PCT, and CRP across day −1 and day 0, their day-to-day ratios (−1/−2 and 0/−1), and their albumin-adjusted ratios. In total, 18 Mann–Whitney U and 18 logistic regression *p*-values were included in the correction. IL-6 on day 0 (both daily values and adjusted) and CRP on day 0 retained significance in both the Mann–Whitney U test and logistic regression. In contrast, the IL-6 level on day −1 lost significance after correction. Details are provided in Appendix A Table A1.

### 3.4. Antibiotic Treatment of Patients Who Do Not Meet the Criteria

Fifteen patients were given antibiotics for VAP without fulfilling the criteria for VAP. Considering those observed with PCT/CRP, 10 patients were treated without fulfilling the criteria for VAP, while under IL-6 observation, five patients were treated (χ^2^(1) = 7.190, *p* = 0.007, V = 0.270). In this group, we administered antibiotics 5.07 (SD 1.75) days after admission. Three of these patients had purulent secretions, whereas five had purulent secretions in the nonVAP group (χ^2^(1) = 5.412, *p* = 0.020, V = 0.255). None of the patients had infiltrates on the day when antibiotic therapy was started. On that day, differences between groups did not reach statistical significance (*p* > 0.05), although a numerical trend was observed in IL-6, CRP, the PaO_2_/FiO_2_ ratio, WBC count, and temperature. The details are provided in Table 5.

## 4. Discussion

Biomarkers play an important role in the early identification of infections, the timely initiation of antimicrobial therapy, and the evaluation of treatment response and duration. However, they seem inconsistent in diagnosing VAP. The predictive value of biomarkers in the context of VAP, therefore, remains a subject of ongoing investigation [24].

We designed an exploratory proof-of-concept study addressing one primary research question. We wanted to determine whether day-to-day ratios, or their ratios to albumin, had greater predictive value for VAP than the daily biomarker measures alone.

Given that inflammatory responses differ greatly between individuals [18] and that they downregulate albumin synthesis [21], we assumed that day-to-day and albumin ratios may increase diagnostic value for VAP.

Among all investigated biomarkers, IL-6 and its day-to-day ratio demonstrated the strongest and most consistent associations with VAP, with high effect sizes of 0.4–0.6 and AUC values above 0.8. Interestingly, IL-6 and its ratios were able to differentiate between VAP and nonVAP patients one day before VAP recognition. Despite this biological rationale, we could not observe any advantage in day-to-day ratios or in albumin adjustment. As for the day-to-day ratios, variability depends strongly on how stable the previous measurement was. Since IL-6 changes very quickly after even a minor stimulus, it can hide real differences rather than highlight them. For albumin adjustment, interindividual differences, e.g., non-inflammatory factors, might be a reason.

In contrast, PCT and CRP, as well as their respective albumin ratios and day-to-day changes, showed weaker significance in differentiating VAP patients from controls with only moderate predictive accuracy. Overall, we did not observe any improvement in discriminatory ability when the ratios were applied.

This might be due to a delayed response of CRP to an inflammatory stimulus. In terms of the values after the onset of VAP, CRP was still able to discriminate between VAP and nonVAP patients, especially on day 1 and day 2, when the median values were even higher than those on day 0 (14.3 mg/dL and 15.85 mg/dL vs. 12.60 mg/dL, respectively). For PCT, we detected very low values in all patients. One explanation might be that PCT responds differently depending on the type of bacteria, the infection site, and a certain delay [13]. However, our expectation of achieving more robust results when adjusting for albumin was not confirmed. This may be due to interindividual variability in albumin levels, which are affected by factors such as nutritional status.

To our knowledge, this was the first study to investigate day-to-day values and albumin ratios in VAP. In former studies, a combination of CRP and IL-6 showed the highest AUC of 0.79 compared to a wide panel of biomarkers for VAP [25]. Other investigators found that the CRP-to-albumin ratio was associated with community-acquired pneumonia in children [26]. In recent years, Pancreatic Stone Protein (PSP) and Pentraxin 3 (PT3) have emerged as biomarkers of interest in pneumonia. In a study by Ceccato et al., the kinetics of PSP were evaluated in serial measures, but no improvement was observed compared to PCT or CRP alone in the differentiation of VAP [25]. Also, Pentraxin 3 did not show superiority over the standard biomarker [26]. Therefore, we investigated the two most common biomarkers, PCT and CRP, in comparison with IL-6.

Second, we differentiated suspected VAP patients from microbiologically confirmed patients. In one-third of VAP patients, we could not identify a bacterium in tracheal or bronchial specimens.

In a recent update, the U.S. Centers for Disease Control (CDC) proposed differentiation. It includes worsening of oxygenation, a condition that is referred to as a “ventilator-associated event” (VAE). In addition to signs of inflammation plus the initiation of antimicrobial therapy, the CDC has proposed the name “ventilator-associated condition” (VAC). Upon microbiological confirmation, the CDC defines it as “infection-related ventilator-associated complication” (IVAC). This definition and the differentiations are, however, not intended for clinical diagnosis but rather for disease surveillance [27]. For clinical purposes, it is important to decide whether a patient requires directed antibiotic treatment. Therefore, our pragmatic approach was close to the CDC’s definition of VAC. Unless bedside techniques are available to identify bacterial infection faster and reliably, we believe that antibiotic treatment should not depend on microbiological confirmation. To address the problem of early bacterial detection, electronic nose sensor arrays and/or bed site nanopore sequencing might be promising approaches [28,29].

Third, our study revealed that some patients who received antibiotics did not meet the VAP criteria. This was particularly common in the CRP/PCT observation period compared with the IL-6 group (*n* = 10 (66.7%) vs. n = 5 (33.3%)). In previous studies, PCT was the focus of extensive research to guide the initiation or discontinuation of antibiotic therapy [13,30]. Our findings suggest that IL-6, known for its greater sensitivity and faster response time [10], may help refine antibiotic stewardship in ICUs.

### Limitations

This study has several significant limitations.

First, the sample size is limited. This may have prevented the identification of further significant differences. Also, this was designed as a monocentric proof-of-concept study; the generalizability of our findings is limited. However, the monocentric design ensured standardized diagnostic and therapeutic procedures, thereby reducing inter-institutional bias and potentially strengthening the validity of our results.

Another limitation is that due to a change in institutional protocols, IL-6 and PCT/CRP were not measured simultaneously across all patients. This led to two distinct cohorts, which may have introduced a comparative bias that should be considered when interpreting the results, even though the baseline characteristics, including the SAPS score, did not differ.

Another relevant limitation is that, although IL-6 appears to be the most promising marker, its availability is limited due to its relatively high cost. It might therefore be useful to conduct prospective studies that assess cost-effectiveness and its effect on antibiotic prescription frequency, as well as other specific outcome data.

## 5. Conclusions

In this exploratory matched case–control study, we identified IL-6, including its day-to-day dynamics and albumin-adjusted values, as the most robust and consistent biomarker associated with VAP in neurosurgical ICU patients. These findings may be of interest for the development of automated predictive or AI-based models and should be validated in a large-scale, multicenter study.

## Figures and Tables

**Table 1:** PCT levels and ratios on day −1 and day 0.

Variable	nonVAP Median (IQR)	mcVAP and suspVAP Median (IQR)	MW *p*-Value (W; r-Effect Size)	AUC	OR (95% CI)	LR *p*-Value
Day −1						
PCT in ng/mL	*n* = 39	*n* = 44				
Day −1	0.16 (0.32)	0.24 (0.47)	0.151 (616.00; 0.189)	0.595	1.017 (0.969–1.068)	0.355
Day −1/−2 Ratio	0.96 (0.38)	1.00 (1.42)	0.177 (321.00; 0.209)	0.605	1.231 (0.823–1.842)	0.065
PCT to Albumin Ratio	17.00 (29.30)	22.77 (37.36)	0.775 (491.00; 0.043)	0.521	1.033 (0.924–1.155)	0.463
Day 0						
PCT in ng/mL	*n* = 31	*n* = 42				
Day 0	0.11 (0.19)	0.24 (0.34)	0.004 * (616.00; 0.414)	0.707	1.010 (0.978–1.043)	0.461
Day 0/−1 Ratio	0.757 (0.28)	1.00 (0.44)	0.126 (241.00; 0.252)	0.626	1.428 (0.516–3.952)	0.492
PCT to Albumin	0.04 (0.05)	0.08 (0.15)	0.005 (192.50; 0.474)	0.746	1.062(0.116–15.688)	0.016

PCT levels, day-to-day ratios, and albumin ratios between nonVAP patients and VAP patients (mcVAP and suspVAP) on days −1 and 0 of VAP occurrence. The results are shown as medians with interquartile ranges (IQRs). The Mann–Whitney U test (MW *p*-value) indicates group differences, with effect sizes (r) reported. Predictive values are reported as odds ratios (ORs) with 95% confidence intervals (CIs), *p*-values (LR *p*-values), and area under the curve (AUC) values. * indicates statistical significance at *p* < 0.05.

**Table 2:** Baseline characteristics.

Baseline	nonVAP *n* = 68	mcVAP/suspVAP *n* = 68	ABnonVAP *n* = 15	Total *n* = 171
Age (years) (SD)	67.22 (14.23)	65.0 (14.39)	60.0 (9.43)	66.4 (13.93)
Sex F/M (% of total)	36/32 (26/15)	21/47 (14/32)	14/11 (7/6)	92/101 (48/52)
Length of stay in hours (SD)	399.81 (360.13)	599.46 (335.57)	532.89 (424.82)	502.29 (367.60)
Hours of ventilation in hours (SD)	198.49 (165.23)	380.10 (225.32)	366.20(370.93)	297.59 (236.05)
SAPS on admission [SD]	38.71 (10.94)	42.55 (10.02)	41.22 (17.32)	40.30 (11.11)
Diagnosis (% of Total)				
Intracerebral bleeding	55 (36.4)	58 (38.3)	15 (9.9)	128 (84.8)
Traumatic head injury	4 (2.6)	3 (2.0)	0 (0)	7 (4.6)
Cerebral tumor	3 (2.0)	0 (0.5)	0 (0)	3 (2.0)
Malignant infarction	6 (4.0)	7 (4.6)	0 (0)	13 (8.6)
Comorbidity (% of Total)				
Cardiovascular disease	44 (29.1)	45 (29.8)	9 (6.0)	98 (64.9)
Respiratory disease	4 (2.4)	3 (2.0)	3 (2.0)	10 (6.6)
Kidney disease	1 (0.7)	3 (2.0)	2 (1.3)	6 (4.0)
Substance abuse	5 (3.3)	9(6.0)	2 (1.3)	16 (10.6)
Other	3(2.0)	11 (7.3)	3 (2.0)	17 (11.3)
Baseline on day of ICU Admission				
WBC (IQR)	11.51 (6.280)	10.34 (8.29)	11.45 (7.29)	10.8 (6.74)
IL-6 (IQR)	40.80 (53.40)	34.05 (97.73)	34.85 (22.05)	40.80 (69.30)
PCT (IQR)	0.3 (0.35)	0.11 (0.06)	0.07 (0.05)	0.11 (0.17)
CRP (IQR)	0.20 (0.95)	0.30 (2.15)	0.45 (1.05)	0.20 (1.55)

Baseline characteristics, diagnoses, comorbidities, and operative procedures of the study population. Data are presented as the means (standard deviations) for continuous variables and daily numbers (percentages of total) for categorical variables. IL-6, PCT, and CRP are presented within the IL-6 and PCT/CRP groups.

**Table 3:** IL-6 levels and ratios on day −1 and day 0.

Variable	nonVAP Median (IQR)	mcVAP and suspVAP Median (IQR)	MW *p*-Value (W; r-Effect Size)	AUC	OR (95% CI)	LR *p*-Value
Day −1						
IL-6 in pg/mL	*n* = 40	*n* = 52				
Day −1	46.35 (63.10)	63.10 (71.00)	*p* = 0.040 * (739.50; 0.255)	0.627	1.001 (0.998–1.004)	0.545
Day −1/−2 Ratio	0.93 (1.42)	1.84 (1.92)	*p* = 0.013 * (627.00; 0.313)	0.656	1.289 (0.970–1.713)	0.059
IL-6-Albumin Ratio	17.00 (29.30)	22.77 (37.36)	*p =* 0.054 (551.50; 0.257)	0.629	1.004 (0.995–1.013)	0.415
Day 0						
IL-6 in pg/mL	*n* = 39	*n* = 53				
Day 0	35.40 (35.80)	109 (180.25)	*p* < 0.001 * (359.50; 0.631)	0.816	1.012 (1.004–1.020)	*p* < 0.001 *
Day 0/−1 Ratio	0.74 (0.81)	1.42 (1.85)	*p* < 0.001 * (500.00; 0.452)	0.726	2.660 (1.390–5.091)	*p* < 0.001 *
IL-6 to Albumin Ratio	12.09 (14.72)	41.48 (65.07)	*p* < 0.001 * (151.50; 0.257)	0.864	1.065 (1.024–1.109)	*p* < 0.001 *

IL-6/PCT/CRP levels, day-to-day ratios, and albumin ratios between nonVAP patients and VAP patients (mcVAP and suspVAP) on days −1 and 0 of VAP occurrence. The results are shown as medians with interquartile ranges (IQRs). The Mann–Whitney U test (MW *p*-value) indicates group differences, with effect sizes (r) reported. Predictive values are reported as odds ratios (ORs) with 95% confidence intervals (CIs), *p*-values (LR *p*-values), and area under the curve (AUC) values. * indicates statistical significance at *p* < 0.05.

**Table 4:** CRP levels and ratios on day −1 and day 0.

Variable	nonVAP Median (IQR)	mcVAP and suspVAP Median (IQR)	MW *p* Value (W; r-Effect Size)	AUC	OR (95% CI)	LR *p*-Value
Day −1						
CRP in mg/L	*n* = 34	*n* = 41				
Day −1	7.60 (7.50)	9.65 (12.38)	0.207 (517.00; 0.175)	0.588	1.058 (0.989–1.132)	0.084
Day −1/−2 Ratio	1.09 (1.12)	1.42 (2.58)	0.177 (242.00; 0.224)	0.612	1.005 (0.956–1.058)	0.835
CRP to Albumin Ratio	3.02 (3.38)	3.07 (5.52)	0.648 (435.50; 0.069)	0.535	0.982 (0.936–1.030)	0.372
Day 0						
CRP in mg/L	*n* = 32	*n* = 37				
Day 0	7.40 (7.95)	12.85 (8.55)	0.015 * (342.00; 0.351)	0.676	1.093 (1.009–1.183)	0.012 *
Day 0/−1 Ratio	0.94 (0.76)	1.07 (0.78)	0.138 (184.50; 0.262)	0.631	1.028 (0.909–1.162)	0.654
CRP to Albumin	3.19 (2.93)	4.25 (2.96)	0.112 (200.00; 0.273)	0.636	1.131 (0.926–1.382)	0.199

CRP levels, day-to-day ratios, and albumin ratios between nonVAP patients and VAP patients (mcVAP and suspVAP) on days −1 and 0 of VAP occurrence. The results are shown as medians with interquartile ranges (IQRs). The Mann–Whitney U test (MW *p*-value) indicates group differences, with effect sizes (r) reported. Predictive values are reported as odds ratios (ORs) with 95% confidence intervals (CIs), *p*-values (LR *p*-values), and area under the curve (AUC) values. * indicates statistical significance at *p* < 0.05.

**Table 5:** Comparison of VAP vs. ABnonVAP.

Variable	nonVAP (Median, IQR)	ABnonVAP (Median, IQR)	*p*-Value (W-Value, r-Effect Size)
Day 0	*n* = 68	*n* = 15	
IL-6 (pg/mL)	35.40 (35.80)	42.80 (42.40)	*p* = 0.941 (W = 95.00, r = 0.026)
PCT (ng/mL)	0.11 (0.19)	0.11 (0.15)	*p* = 0.738 (W = 167.50, r = 0.069)
CRP (mg/L)	7.40 (7.95)	12.75 (6.03)	*p* = 0.071 (W = 95.00, r = 0.387)
WBC (×10^9^/L)	9.30 (4.56)	10.10 (4.15)	*p* = 0.569 (W = 358.50, r = 0.098)
Temp (°C)	37.20 (0.80)	37.50 (0.45)	*p* = 0.412 (W = 414.50, r = 0.136)
PaO_2_/FiO_2_	347.00 (128.00)	308.00 (147.00)	*p* = 0.633 (W = 366.00, r = 0.084)

Comparison of relevant markers between nonVAP patients and those who were treated for VAP without fulfilling the criteria. The values are presented as medians (IQRs).

## Data Availability

The datasets generated and analyzed during this study are not publicly available due to institutional restrictions and data protection regulations. Anonymized data may be made available from the corresponding author upon reasonable request and with permission from the responsible institutions.

## Abbreviations

ABnonVAP	Patients treated with antibiotics for VAP without fulfilling diagnostic criteria
AI	Artificial Intelligence
AUC	Area Under the Curve
CDC	Centers for Disease Control and Prevention
CI	Confidence Interval
CRP	C-reactive Protein
D0	Study days relative to VAP diagnosis
HRG	Histidine-Rich Glycoprotein
ICU	Intensive Care Unit
IL-6	Interleukin-6
IQR	Interquartile Range
IVAC	Infection-Related Ventilator-Associated Complication
LR *p*-value	Logistic Regression *p*-value
mcVAP	Microbiologically confirmed VAP
MW *p*-value	Mann–Whitney U test *p*-value
NonVAP	Patients without VAP (control group)
OR	Odds Ratio
PCT	Procalcitonin
ROC	Receiver Operating Characteristic (curve)
SAPS	Simplified Acute Physiology Score
SD	Standard Deviation
suspVAP	Suspected but not microbiologically confirmed VAP
Temp	Body Temperature (°C)
US	United States
VAC	Ventilator-Associated Condition
VAE	Ventilator-Associated Event
VAP	Ventilator-Associated Pneumonia
WBC	White Blood Cell count
PaO_2_/FiO_2_ ratio (P/F ratio)	Ratio of arterial oxygen partial pressure to fraction of inspired oxygen

## Appendix A

**Table A1:** Matching results.

Characteristic	Unmatched mcVAP + suspVAP	Unmatched nonVAP Controls	*p*-Value	Matched mcVAP + suspVAP	Matched nonVAP Controls	*p*-Value
Sample size (*n*)	73	83		68	68	
SAPS Score (SD)	41.68 (11.07)	38.45 (10.78)	0.079	42.55 (10.92)	38.89 (10.94)	0.064
Age (SD)	65.36 (14.01)	67.36 (13.93)	0.372	65.0 (14.39)	67.22 (14.30)	0.368

SAPS score and age before and after matching between VAP patients (mcVAP + suspVAP) and nonVAP controls. The values are given as the means (SDs).

**Table A2:** Number of patients within the biomarker groups.

Cohort	Controls	mcVAP	suspVAP	ABnonVAP	Total
IL-6 (%)	52 (31.1)	32 (19.2)	20 (12.0)	5 (3.0)	109 (65.3)
PCT/CRP (%)	16 (9.6)	13 (7.8)	3 (1.8)	10 (6.0)	42 (25.1)
Total	68 (40.7)	45 (26.9)	23 (13.8)	15 (9.0)	151 (100.0)

Distribution of patients across biomarker groups (IL-6 vs. PCT/CRP) and clinical VAP groups (controls, mcVAP, suspVAP, ABnonVAP). The percentages refer to the proportion of all included patients.

**Table A3:** Benjamini–Hochberg correction.

Variable	MW_*p*	LR_*p*	MW_*p*_adj	LR_*p*_adj
CRP Day −1	0.177	0.835	0.212	0.835
Day −1/−2 Ratio	0.648	0.372	0.686	0.5904
CRP-Albumin-Ratio Day −1	0.648	0.372	0.686	0.5904
CRP Day 0	0.015	0.012	0.039	0.0432
Day 0/−1 Ratio	0.138	0.654	0.207	0.692470588
CRP to Albumin Day 0	0.112	0.199	0.202	0.44775
PCT Day −1	0.151	0.355	0.209	0.5904
PCT Day −1/−2 Ratio	0.177	0.065	0.212	0.167142857
PCT-Albumin-Ratio Day −1	0.775	0.463	0.775	0.5904
PCT Day 0	0.004	0.436	0.014	0.5904
PCT Day 0/−1 Ratio	0.126	0.492	0.206	0.5904
PCT to Albumin Day 0	0.005	0.018	0.014	0.036
IL-6 Day −1	0.040	0.545	0.090	0.613125
IL6 Day −1/−2 Ratio	0.013	0.059	0.039	0.167142857
IL-6-Albumin-Ratio Day −1	0.054	0.415	0.108	0.5904
IL-6 Day 0	<0.001	<0.001	0.006	0.006
IL-6 Day 0/−1 Ratio	<0.001	<0.001	0.006	0.006
IL-6 to Albumin Day 0	<0.001	<0.001	0.006	0.006

*p*-value of Mann–Whitney U tests (MW_*p*), logistic regression analyses (LR_*p*), and Benjamini–Hochberg corrected *p*-values (MW_*p*_adj and LR_*p*_adj). The Benjamini–Hochberg procedure was applied only to the associated *p*-values.
